# Microbial fuel cell-assisted utilization of glycerol for succinate production by mutant of *Actinobacillus succinogenes*

**DOI:** 10.1186/s13068-021-01882-5

**Published:** 2021-01-15

**Authors:** Tianwen Zheng, Bin Xu, Yaliang Ji, Wenming Zhang, Fengxue Xin, Weiliang Dong, Ping Wei, Jiangfeng Ma, Min Jiang

**Affiliations:** 1 State Key Laboratory of Materials-Oriented Chemical Engineering, College of Biotechnology and Pharmaceutical Engineering, Nanjing Tech University, Puzhu South Road 30#, Nanjing, 211800 P. R. China; 2 Jiangsu National Synergetic Innovation Center for Advanced Materials (SICAM), Nanjing Tech University, Nanjing, 211800 P. R. China

**Keywords:** Microbial fuel cell, *Actinobacillus succinogenes*, Succinate, Glycerol utilization, ARTP mutagenesis

## Abstract

**Background:**

The global production of glycerol is increasing year by year since the demands of biodiesel is rising. It is benefit for high-yield succinate synthesis due to its high reducing property. *A. succinogenes*, a succinate-producing candidate, cannot grow on glycerol anaerobically, as it needs a terminal electron acceptor to maintain the balance of intracellular NADH and NAD^+^. Microbial fuel cell (MFC) has been widely used to release extra intracellular electrons. However, *A. succinogenes* is a non-electroactive strain which need the support of electron shuttle in MFC, and pervious research showed that acid-tolerant *A. succinogenes* has higher content of unsaturated fatty acids, which may be beneficial for the transmembrane transport of lipophilic electron shuttle.

**Results:**

MFC-assisted succinate production was evaluated using neutral red as an electron shuttle to recover the glycerol utilization. First, an acid-tolerant mutant JF1315 was selected by atmospheric and room temperature plasma (ARTP) mutagenesis aiming to improve transmembrane transport of neutral red (NR). Additionally, MFC was established to increase the ratio of oxidized NR to reduced NR. By combining these two strategies, ability of JF1315 for glycerol utilization was significantly enhanced, and 23.92 g/L succinate was accumulated with a yield of 0.88 g/g from around 30 g/L initial glycerol, along with an output voltage above 300 mV.

**Conclusions:**

A novel MFC-assisted system was established to improve glycerol utilization by *A. succinogenes* for succinate and electricity production, making this system as a platform for chemicals production and electrical supply simultaneously.

## Background

Glycerol, in most case, is obtained during either hydrolytic synthesis of fatty acid (soap) or biodiesel production. The global annual production of glycerol is estimated to reach 4.2 million tons in 2020 with the increasing demands of biodiesel (1 ton glycerol generation of every 9 tons biodiesel), while the demand is less than 3.5 million tons [1]. Thus, glycerol becomes surplus and it is urgent to explore novel approaches to utilize glycerol for value-added chemicals productions. Glycerol offers a wide range of applications due to its unique property, renewability and availability in the current market [2]. As microbial biomass (oxidation state, – 0.4) [3] is less reduced than glycerol (oxidation state, – 0.67), bacteria growing with glycerol would excrete products, such as 1, 3-propanediol (oxidation state, – 1.33), with a redox state lower than that of glycerol to ensure redox balance. Otherwise, those products, such as malate (oxidation state + 1), succinate (oxidation state + 0.5), need extra electron acceptor to balance the redox state [4].

**Fig. 1:**
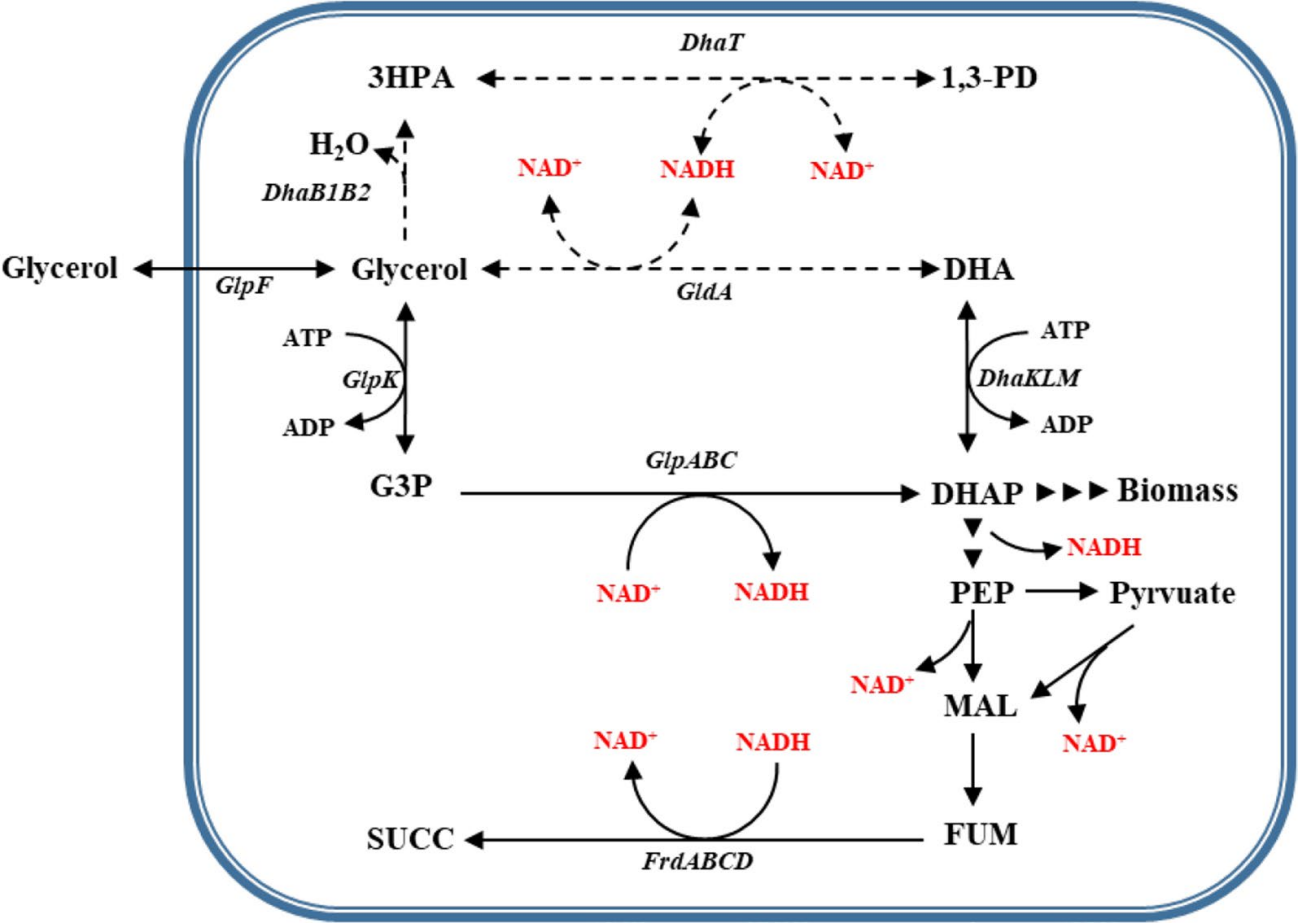
Metabolic pathways involved in glycerol utilization by *A. succinogenes* NJ113. Dashed lines: 1,3-PD pathway (absent in *A. succinogenes). *Black lines: DHAP pathway (present in *A. succinogenes*). Abbreviation: 3HPA, 3-hydroxypropionaldehyde; 1,3-PD, 1,3-propanediol; G3P, glyceraldehyde-3-phosphate; DHA, dihydroxyacetone; DHAP, dihydroxyacetone phosphate; FUM, fumarate; SUCC, succinate. Adapted from [4]

*Actinobacillus succinogenes*, isolated from rumen, can produce high level of succinate from a wide range of carbon substrates [5]. However, *A. succinogenes* have poor glycerol utilization ability due to the redox unbalance [4]. As illustrated in Fig. 1, the reduced property of succinate is consistent with glycerol. However, nearly 14% glycerol flowed to biomass. During the growth stage, one NADH is generated for one glycerol entering glycolysis and recycling of NADH can be accomplished by reducing glycerol to 1,3-propanediol (Fig. 1 dashed lines). Unfortunately, *A. succinogenes* lacks such 1,3-propanediol metabolic pathway, leading to the accumulation of NADH due to that biomass is less reduced than glycerol. Thus, the cell growth was inhibited with glycerol as the sole carbon source [6]. To maintain the balance of redox state, additional electron acceptors were supplied, such as dimethyl sulfoxide (DMSO) and nitrate [4]. Durnina et al. adopted the micro-aerobic fermentation by supplementing small amount of oxygen to oxidize the NADH through respiratory chain [7]. Schindler et al. introduced heterologous NADH-dependent pathways to enhance the oxidation of NADH and recover the balance of NADH/NAD^+^ [4].

All these strategies promoted cell growth and glycerol utilization, but the energy in NADH was wasted. Microbial fuel cells have been widely used for electricity generation with various microorganisms as biocatalyst, such as *Shewanella* *spp*. and *Geobacter sulfurreducens* [8–10]. In those MFCs, electrons generated from intracellular metabolism can be transferred to anode electrode directly via nanowires and outer membrane proteins or indirectly by electron shuttles. Then, electrons are finally transferred to cathode electrode via external circuit with the generation of electricity power [11]. As a result, the electrons in the excess NADH can be effectively utilized for the current generation and power output in MFC. Also, MFC has been adopted for production of oxidized products along with the power output. Bursac and colleagues used an engineered *Shewanella oneidensis* to produce acetoin from lactate in an anode-assisted unbalanced fermentation system. The surplus electrons can be transferred to the poised electrode directly, and acetoin become the sole end product and byproducts were eliminated [12]. Anode-assisted acetoin production was also conducted by Forster and colleagues in an engineered *Escherichia coli*. The byproducts were also dramatically reduced, and the yield of acetoin reached 79% of the theoretical maximum [13].

Unlike electrochemical active bacteria, there are no nanowires or outer membrane appendages in *A. succinogenes*, which means electrons cannot be transferred between bacteria and electrodes directly. Thus, the electrochemical inactive strains need the support of electron shuttles to interact with electrodes [14, 15]. It was reported that *A. succinogenes* can capture electrons from cathode electrode with the electron shuttle neutral red (NR), although the mechanism of electron transfer has not been elucidated [16]. Previous studies reported that passive diffusion of lipophilic compounds, such as NR, can be improved when the degree of unsaturation of some defined phospholipid molecular species was increased [17], and acid-resistant *A. succinogenes* had higher content of unsaturated fatty acids [18].

In this study, ARTP mutagenesis [19] was carried out to select acid-tolerant *A. succinogenes* mutant to improve the transmembrane transport of NR. Then, glycerol utilization and cell growth along with electricity output were investigated in MFC system, aiming to establish a novel anode platform with chemicals production and electrical supply simultaneously.

## Results

### Screening of electron shuttles to facilitate glycerol utilization by *A. succinogenes* NJ113

**Fig. 2:**
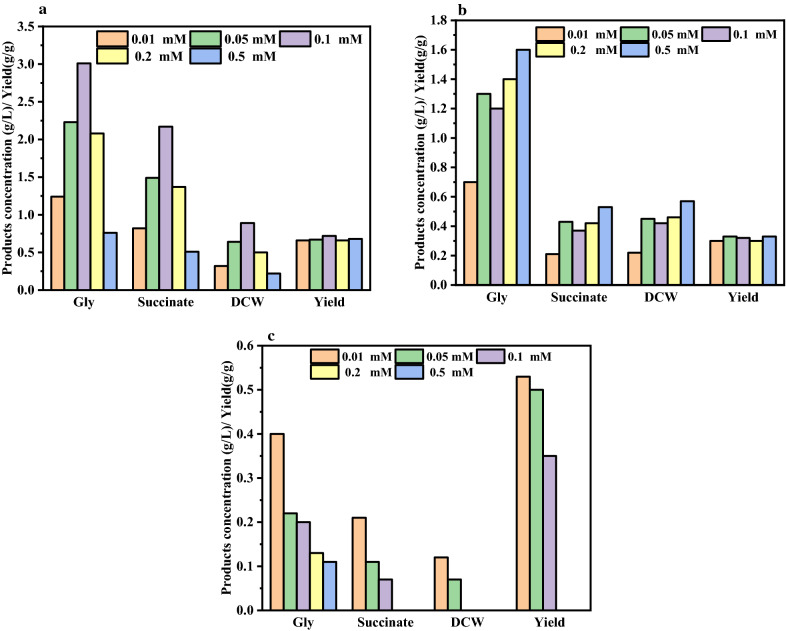
Screening of electron shuttlers to facilitate glycerol utilization by *A. succinogenes* NJ113. a Fermentations in sealed bottles by NJ113 supplemented with different concentrations of neutral red; **b** Fermentations in sealed bottles by NJ113 supplemented with different concentrations of riboflavin; **c **Fermentations in sealed bottles by NJ113 supplemented with different concentrations of methylene blue

First, glycerol utilization by *A. succinogenes* NJ113 was investigated in sealed bottles supplemented with three commonly used electron shuttles: neutral red, riboflavin and methylene blue. As shown in Fig. 2, the highest dry cell weight (DCW) and glycerol consumption were achieved by supplemented with 0.1 mM NR. The DCW reached 0.89 g/L, and 3.01 g/L of glycerol was consumed with 2.17 g/L succinate accumulated after 72 h (Fig. 2a). Other two electron shuttles also showed the ability to promote glycerol utilization of NJ113. However, the glycerol consumption and the succinate production with riboflavin were lower than those supplemented with NR (Fig. 2b). Figure 2c shows that methylene blue was toxic to NJ113 even though concentration was below 0.1 mM.

**Fig. 3:**
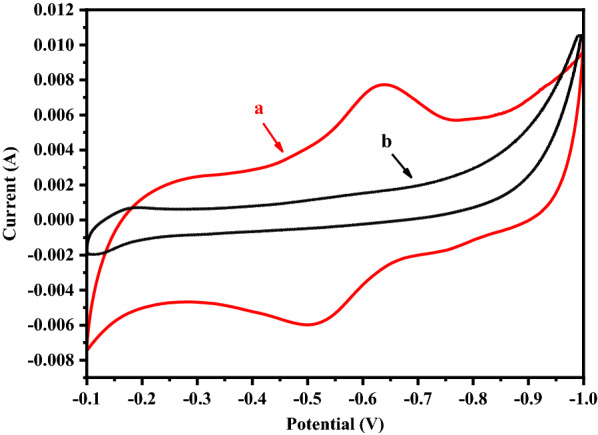
Cyclic voltammetry curve of glycerol fermentation in MFC system by A. succinogenes NJ113 with or without NR.** a** CV curve of glycerol fermentation with 0.1 mM NR;** b** CV curve of glycerol fermentation without NR

**Fig. 4:**
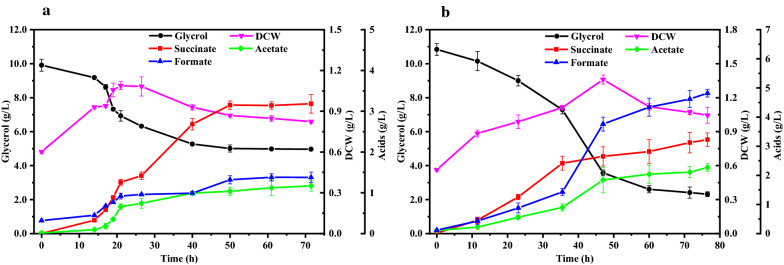
Glycerol utilization by *A. succinogenes* NJ113 in MFC system without and with 0.2 V anode potential. **a **Fermentation in MFC by NJ113 without potential applied; **b** Fermentation in MFC by NJ113 with 0.2 V potential applied in anode electrode

Considering that 0.1 mM NR might be insufficient but NR higher than 0.1 mM was toxic to NJ113 (Fig. 2), anaerobic fermentation was further carried out in MFC to oxidize the reduced NR. Figure 3 shows the cyclic voltammetry curve in MFC system with or without NR. The oxidation peak (-0.51 V) and reduction peak (-0.63 V) were consistent with previous report that midpoint of redox potential of NR was -0.56 V (V.S. Ag/AgCl) [13]. This indicated that NR can be adopted as an electron shuttle in this glycerol fermentation medium with unknown electron acceptor, likely derived from the yeast extract. As a result, glycerol consumption was increased from 3.01 to 4.94 g/L in MFC (Fig. 4), which might be due to the supplement of sufficient oxidized NR. However, the glycerol consumption rate was still very low. Thus, fermentations were further carried out in MFC with a 0.2 V potential applied in anode electrode to investigate the effects of promoted anode oxidized reaction. As a result, the glycerol consumption was increased to 8.52 g/L. However, the yield of succinate was reduced from 0.64 to 0.38 g/g with more accumulation of formate.

### Mutation and selection of acid-resistant *A. succinogenes*

Glycerol utilization by *A. succinogenes* NJ113 can be promoted by accelerating electron transfer rate with anode potential, but the carbon flux was redirected to relative oxidized byproducts. Pervious research showed that *A. succinogenes* gained a better pH tolerance by increasing the content of unsaturated fatty acids. Thus, we assumed that this mutant may have improved ability of transmembrane transport of lipophilic neutral red. Here, we adopted ARTP strategy to select acid-resistant mutants. First, *A. succinogenes* NJ113 was treated by ARTP mutagenesis for 30 s according to the lethal curve (Additional file 1: Figure S1). After several rounds of ARTP treatment and acid stress test, five mutants of *A. succinogenes* NJ113 were obtained which could grow on the acidic agar plates (pH 5.8). To investigate the growth and metabolism performance, all five mutants were tested by anaerobic fermentation in sealed bottles at pH 5.8 with 10 g/L glucose. As shown in Table 1, four mutants (JF1311, JF1313, JF1315 and JF1319) had better performance of glucose utilization and succinate production compared to that of *A. succinogenes* NJ113. Among them, strain JF1315 produced the highest 5.93 g/L succinate with the yield of 0.55 g/g, which is 83.33% higher than that of parent strain (0.30 g/g) under similar condition. It indicated that *A. succinogenes* JF1315 had the best acid-resistant ability.

**Table 1:** Cell growth and glucose metabolism of *A. succinogenes* in sealed bottles

Strains	Glucose^a^ (g/L)	DCW (g/L)	Succinate (g/L)	Acetate (g/L)	Formate (g/L)	Y_Suc/Glu_^b^ (g/g)
NJ113	5.71 ± 0.07	0.55 ± 0.03	1.73 ± 0.05	1.36 ± 0.03	1.92 ± 0.03	0.30 ± 0.05
JF1311	7.23 ± 0.11	1.19 ± 0.01	2.82 ± 0.07	1.97 ± 0.02	1.76 ± 0.05	0.39 ± 0.09
JF1313	8.97 ± 0.09	0.97 ± 0.01	2.79 ± 0.04	1.59 ± 0.02	1.95 ± 0.01	0.31 ± 0.03
JF1315	10.70 ± 0.15	1.04 ± 0.07	5.93 ± 0.03	1.99 ± 0.07	1.69 ± 0.01	0.55 ± 0.03
JF1317	5.12 ± 0.06	0.57 ± 0.01	1.92 ± 0.07	1.13 ± 0.01	2.03 ± 0.07	0.38 ± 0.02
JF1319	9.55 ± 0.04	0.92 ± 0.05	3.45 ± 0.03	1.37 ± 0.04	1.76 ± 0.05	0.36 ± 0.01

Each value is an average of three parallel replicates and reported as mean ± standard deviation

^a^ Around 10 g/L glucose was supplemented in the fermentation medium, and the values represented the amount of consumed glucose

^b^ Y_Suc/Glu_ represents succinate (Suc) yield on the glucose (Glu)

**Fig. 5:**
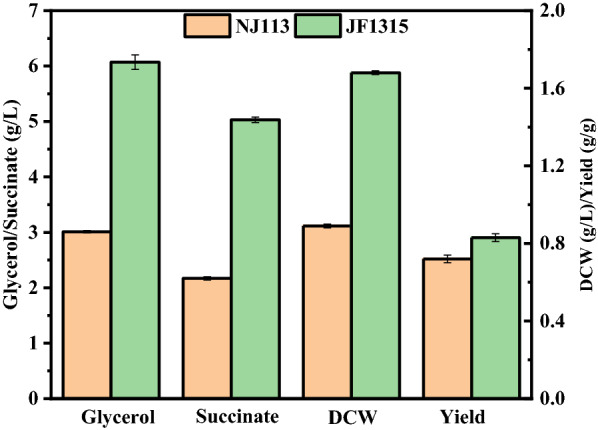
Glycerol utilization by *A. succinogenes* NJ113 and its mutant JF1315 in sealed bottles with 0.1 mM neutral red

As speculated before, the acid-tolerant JF1315 might have improved transmembrane transport of NR. Thus, we further conducted the fermentations in sealed bottles with *A. succinogenes* JF1315 using glycerol as sole carbon source. As summarized in Fig. 5, the DCW and glycerol consumption of *A. succinogenes* JF1315 were 1.68 g/L and 6.07 g/L, increased by 88.8% and 101.6% compared with *A. succinogenes* NJ113, respectively. The enhanced ability of glycerol utilization indicated that the mutant JF1315 had improved bidirectional transportation of NR, although the rate of glycerol consumption was still relatively low and 3.93 g/L glycerol was remaining in the broth after 72 h.

### Enhanced glycerol utilization and succinate synthesis by acid-resistant *A. succinogenes* in MFC

**Fig. 6:**
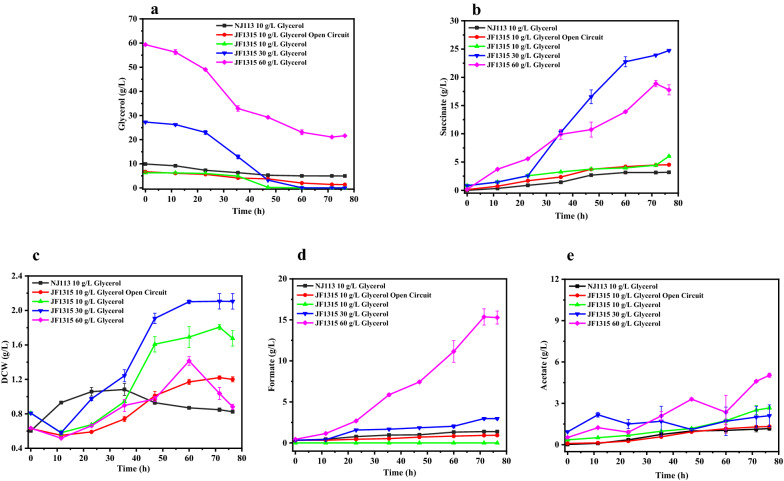
Glycerol utilization and products accumulation by *A. succinogenes* NJ113 and JF1315 in MFC system with different initial glycerol.** a** Glycerol consumption by *A. succinogenes*; **b** Succinate production by *A. succinogenes*; **c** DCW of *A. succinogenes*; **d** Formate production by *A. succinogenes*; **e** Acetate production by *A. succinogenes*

To gain better glycerol utilization and succinate synthesis, mutant JF1315 was further investigated in MFC, and fermentations with different initial glycerol concentrations were carried out. In MFC, glycerol utilization and succinate synthesis were both improved significantly (As shown in Fig. 6). Under low concentration of glycerol (less than 10 g/L), the glycerol was depleted and 5.21 g/L of succinate (0.83 g/g glycerol) was produced with small amounts of byproducts (Fig. 6d, e). In addition, only 5.33 g/L glycerol was consumed when the external resistance was taken away. It indicated effective transfer of electrons in MFC played an important role in glycerol utilization. When the concentration of glycerol was up to 30 g/L, 23.92 g/L succinate accumulated with a yield of 0.88 g/g and the glycerol could be depleted. However, when the glycerol concentration was further increased to 60 g/L, the remaining glycerol was more than 20 g/L and the succinate yield was decreased to 0.57 g/g, which might be due to the remarkable accumulation of 15.28 g/L formate and 5.03 g/L acetate (Fig. 6d, e).

Cell growth was also improved significantly in MFC system with < 30 g/L initial glycerol (Fig. 6c), and the DCW of JF1315 could achieve 2.11 g/L with increment of 93.58% and 25.60% compared with NJ113 in MFC (1.09 g/L) and JF1315 in sealed bottles (1.68 g/L), respectively. However, when initial concentration of glycerol was up to around 60 g/L, cell growth was inhibited at the late stage of anaerobic fermentation, during which high concentration of formate and acetate were accumulated.

### MFC performance and power output

**Fig. 7:**
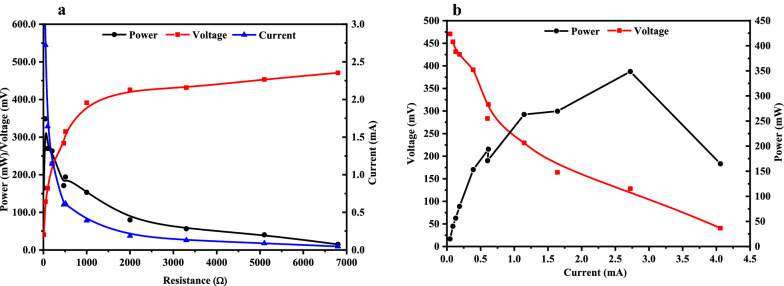
Polarization of the MFC system with *A. succinogenes* JF1315.** a **Relationship between external resistance and the output of power, voltage and current; **b** Relationship between current and the output of voltage and power

To evaluate the performance of MFC system, the polarization curve was made by varying the external resistance. With the increment of the external resistance, cell voltage kept increasing and finally reached 425.5 mV (Fig. 7a), whereas the power output only increased with lower resistance and dropped sharply along with the increment of external resistance. A maximum power output of 348.6 mW was achieved with a current of 2.7 mA and a voltage of 128.0 mV at 47.0 Ω external resistance (Fig. 7b).

**Fig. 8:**
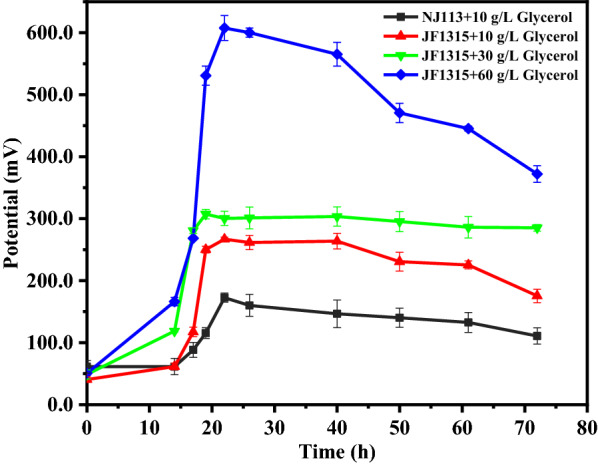
Cell potential output with *A. succinogenes* NJ113 and JF1315 with different initial glycerol in MFC system

As shown in Fig. 8, after *A. succinogenes* NJ113 and JF1315 were inoculated in MFC, cell potential increased to the peak voltage after a short start-up time and then a sustainable power output was generated for at least 48 h with < 30 g/L glycerol. The peak values were 172.6 mV and 266.9 mV in *A. succinogenes* NJ113 and JF1315 with 10 g/L glycerol, respectively. For *A. succinogenes* JF1315, relative constant and high value above 300 mV was obtained for at least 48 h with 30 g/L glycerol. However, potential gradually dropped after the peak value of 607.6 mV when initial glycerol increased up to 60 g/L.

Compared with the long-term power output with 30 g/L glycerol, the stable phase of cell potential did not last long and dropped sharply after 30 h with *A. succinogenes* JF1315 in the presence of 60 g/L glycerol (Fig. 8). It indicated that the cell activity of JF1315 decreased with high concentration of glycerol. In MFC fed with 60 g/L glycerol, more than 15 g/L formate was generated, which might be the main reason leading to the stagnation of cell growth and the decrease of power output.

## Discussion

Generally, glycerol was benefit for high-yield succinate synthesis due to its high reducing property. However, *A. succinogenes* cannot grow on glycerol anaerobically, as it needs a terminal electron acceptor, such as DMSO or nitrate to achieve anaerobic respiration [4]. Thus, we investigated the effects of adding electron shuttle NR on the glycerol consumption in *A. succinogenes* NJ113. As a result, glycerol consumption ability was recovered but the rate was still very low. We proposed that there might be two reasons for limiting the utilization of glycerol: (1) Low ratio of oxidized NR to reduced NR. (2) Poor efficiency of bidirectional transmembrane transfer of NR.

Then, MFC-assisted system for succinate production was established and adopted. However, glycerol cannot be fully utilized by *A. succinogenes* NJ113 even in the presence of sufficient oxidized NR. It indicated that the insufficient oxidized NR was not the sole cause limiting glycerol utilization by *A. succinogenes* NJ113, and further investigation should be carried out to insure whether poor efficiency of bidirectional transmembrane transfer of NR is another significant factor. As *A. succinogenes* NJ113 is a non-electroactive strain, which lacks native transport pathway for transmembrane transfer of electron shuttles. In addition, the out membrane is a relatively low permeable barrier for the transport of electron shuttles [14]. Therefore, the efficiency of bidirectional transmembrane transfer of electron shuttles was seriously restricted [18–20]. Thus, we adopted an effective strategy to enhance the permeability of cell membrane to NR.

Previous studies have introduced or overexpressed out membrane porins, which can form water-filled channels for the diffusion of hydrophilic compounds, to improve the transfer of electron shuttles [14]. Unfortunately, NR is extremely hydrophobic due to the high lipid/water partition coefficient [21]. An alternative strategy is to modify the composition of out membrane to accelerate the diffusion of lipophilic substances. Hagve et al. reported that passive diffusion of lipophilic compounds was improved when the degree of unsaturation of some defined phospholipid molecular species was increased [17]. In addition, elongating the saturated fatty acids or converting them to unsaturated fatty acids is a common bacterial adaptation strategy to resist acid stress [18]. Thus, we aim to enhance the transmembrane transfer of NR in *A. succinogenes* by breeding acid-resistant mutants. Using ARTP treatment and acid stress test, we obtained the acid-tolerant mutant *A. succinogenes* JF1315, which has enhanced glycerol consumption ability in sealed bottles. Consequently, the strategy of breeding acid-resistant mutant is an efficient route to enhance the transmembrane transfer of NR, and it is beneficial for the non-electroactive strain to establish the MFC systems.

Further study with JF1315 in MFC-assisted system showed that cell growth of JF1315 was improved and glycerol could be depleted, when initial glycerol was maintained at a low concentration. It might be due to that MFC system with NR has an acceleration for the intracellular energy metabolism. On one hand, the proton motive force was enhanced through the co-transport of proton and electron across the membrane, and thus ATP synthesis was improved by ATPase. On the other hand, glycerol metabolism via NR made it possible that cytochromes were involved in intracellular electron transfer, and thus more energy (ATP) could be gained for cell growth and cell maintenance [22, 23]. However, when initial concentration of glycerol was up to around 60 g/L, cell growth was inhibited which might be due to the remarkable accumulation of formate. It has been reported that cell growth of *A. succinogenes* was completely inhibited when formate concentration reached 16 g/L [24]. Meanwhile, gradually dropped voltage was gained after the peak value of 607.6 mV which might be also caused by high formate accumulation. Thus, continuous feeding strategy would be adopted with low glycerol condition to gain the maximum yield of succinate with constant power output.

## Conclusions

*Actinobacillus succinogenes* cannot grow on glycerol anaerobically, as it needs a terminal electron acceptor. By the experiments supplemented with NR or carried out in MFC system, we certified that the insufficient oxidized NR was not the sole cause limiting glycerol utilization by *A. succinogenes* NJ113, and we speculate that the inefficiency of bidirectional transmembrane transfer of NR was another key factor.

By several rounds of ARTP and acid stress selection, an acid-tolerant mutant *A. succinogenes* JF1315 was obtained and investigated. High yield of 0.88 g/g succinate could be achieved with around 30 g/L initial glycerol, and relative constant and high value above 300 mV was obtained for at least 48 h. Thus, breeding acid-resistant mutant is an efficient strategy to enhance the transmembrane transfer of NR, and it is beneficial for those non-electroactive strain to conduct experiments in the MFC systems. However, when the initial glycerol up to 60 g/L, remarkable formate and acetate accumulated, and thus gradually dropped voltage was gained after the peak value. Hence, continuous feeding strategy could be adopted to maintain the glycerol at a low concentration.

In conclusion, this study established a novel anode platform based on MFC and published a method to enhance the transmembrane transfer of NR for the non-electroactive *A. succinogenes*, making this system organic acids production and electrical supply simultaneously in future.

## Methods

### Strains and culture conditions

The original strain, *A.* *succinogenes* NJ113 (CGMCC No.11716), was used in this study. The seed medium contained 10.0 g/L glucose, 5.0 g/L yeast extract, 2.5 g/L corn steep liquor, 10.0 g/L NaHCO_3_, 9.6 g/L NaH_2_PO_4_·2H_2_O, 1.0 g/L NaCl and 15.5 g/L K_2_HPO_4_. The solid seed medium contained additional 2% (w/v) agar, and the pH was adjusted to 5.8 by succinic acid for acid-tolerant mutants screening experiments. The fermentation medium contained 10.0 g/L yeast extract, 7.5 g/L corn steep liquor, 1.36 g/L sodium acetate, 1.6 g/L NaH_2_PO_4_·2H_2_O, 0.3 g/L Na_2_HPO_4_·12H_2_O, 3.0 g/L K_2_HPO_4_, 0.2 g/L MgCl_2_·6H_2_O, 0.2 g/L CaCl_2_, and 1.0 g/L NaCl with different concentrations of glucose or glycerol. All the media were sterilized at 121 °C for 15 min, and glucose and glycerol solution were sterilized separately.

The inoculums were prepared in 50 mL medium sealed in anaerobic bottles with the volume of 100 mL. For anaerobic bottle cultivation, exponentially growing cells (10%, v/v) were inoculated into 100 mL sealed anaerobic bottles filled with 30 mL of fermentation medium containing 10 g/L of glucose or different concentrations of glycerol. Magnesium carbonate hydroxide (80% of carbon source, g/L) was added to maintain the pH above 6.4. The anaerobic bottle cultivation was carried out in a rotary shaker at 37 °C and 180 rpm.

### ARTP mutagenesis

The ARTP mutation was implemented with helium as the plasma working gas. The exponentially growing cells were diluted to OD_660_ around 1.0 and 10 μL of the diluted solution was put onto a pre-cooling sterilized stainless steel plate (12.0 mm in diameter). The plates with the cells were then placed into the vessel and treated by helium plasma. The apparatus was operated at the helium gas flow rate of 10 slpm (standard liters per minute) and radiofrequency power input of 100 W with a 2 mm stand-off distance between the torch nozzle exit and the vessel. And plasma treatment time ranged from 0 to 100 s. After mutation, mutagenesis solution was resuspended and coated on solid plates (pH 5.8), and then incubated at 37 °C under anaerobic condition.

### Installation of MFC system

**Fig. 9:**
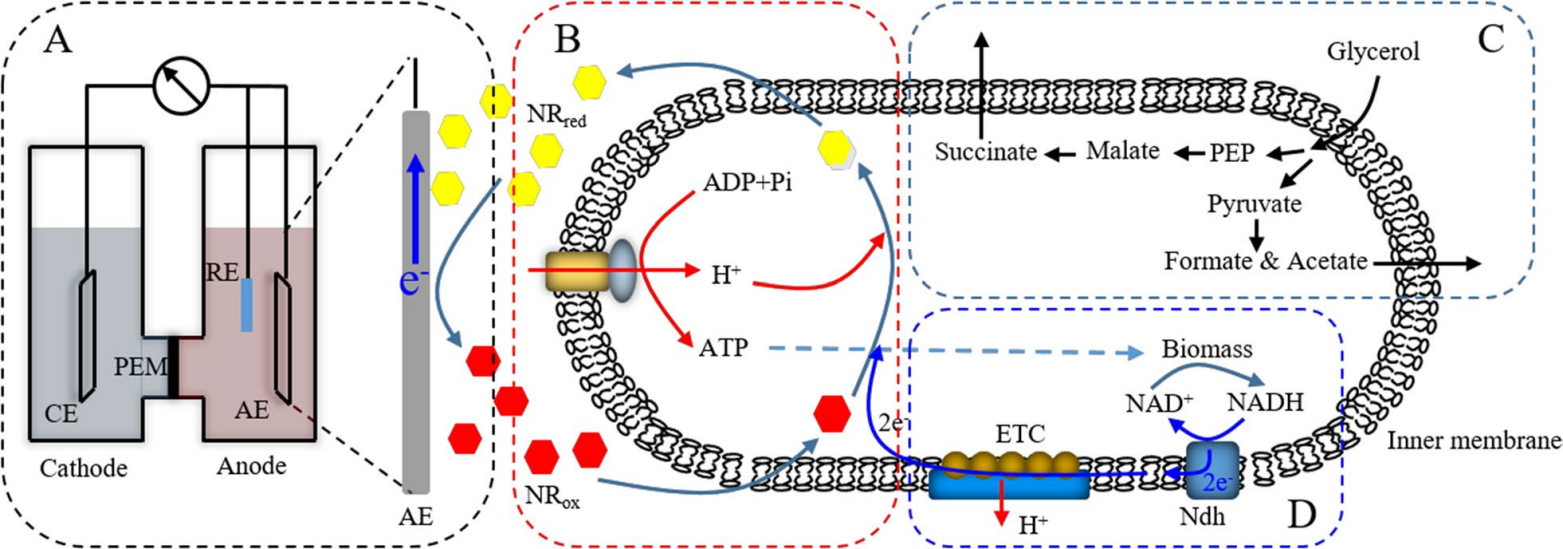
Hypothetical metabolism of *A. succinogenes* in MFC system. Module A: The MFC system for electricity power generation. CE: cathode electrode; AE: anode electrode; RE: reference electrode; PEM: proton exchange membrane; Module B: The mechanism of electron emission via NR and energy metabolism via ATPase. NRred: the reduction state of NR; NRox: the oxidation state of NR; Module C: The pathway of succinate synthesis from glycerol. PEP: phosphoenolpyruvate; Module D: The hypothetical mechanism of electron transfer chain coupled with NADH oxidation and cell growth. ETC: electron transfer chain; Ndh: NADH dehydrogenase

The MFC was established as shown in Fig. 9 and carried out in batch mode by using H-cell model, composed the following two gastight chambers (700 mL), anode and cathode chambers. A Nafion 117 proton exchange membrane (PEM, DuPont, 16.6 cm^2^) was clamped between the two separating chambers. Graphite was used as anode and cathode electrodes (34 cm^2^), and the titanium wire (McMaster-Carr, 0.032 cm in diameter) was used to connect the electrodes for the external circuit. Before and after experiment, the electrodes and PEM were pretreated as Bond [25] and Aulenta [26] said, respectively. A reference electrode (Ag/AgCl sat. KCl, 0.195 V vs SHE, CH Instruments, China) was placed in anode chamber. The potentials mentioned in this study were relative to Ag/AgCl reference electrode.

### Operation and fermentation in MFC

The body of MFC system was installed and autoclaved in 121 °C for 20 min. The anode chamber was filled with 500 mL autoclaved fermentation medium (containing 0.1 mM NR), and the cathode chamber was filled with 500 mL autoclaved Na-phosphate buffer (100 mM, pH 6.8) with NaCl (100 mM). Approximately, 50 mL of fresh harvested early stationary culture was inoculated into the anode chamber (10%, v/v) with sterile CO_2_ purged continuously to achieve an oxygen-free environment. Meanwhile, sterile air was purged into the cathode chamber to supply oxygen for the electrochemical reaction. After inoculation, the reactor was incubated at 37 °C and the two chambers were connected with external resistance. Sodium carbonate solution (15%, w/v) was used to maintain the pH of broth above 6.8.

Cell potential was detected using a digital multimeter (VICTOR 86D, Shenzhen Victor HI-Tech Co., Shenzhen, China). To obtain a polarization curve, the external resistance was varied over a range of 10 Ω to 51 kΩ and the voltage at each resistance was recorded when the voltage output approached the steady-state. Cyclic voltammetry was carried out in the three electrodes arrangement using a multichannel potentiostat (CHI 1000C, China) with the scan rate at 5 mV/s from – 0.1 to – 1.0 V.

## Sample analysis

DCW was computed from a curve relating optical density at 660 nm (OD_660_). OD_660_ of 1.0 represented 0.52 g of dry weight per liter. Organic acids, glucose and glycerol were determined by HPLC (P680 pump, Dionex, USA) equipped with UV and refractive index (RI) detectors. An ion-exclusion Bio-Rad Aminex HPX 87H (7.8 mm × 300 mm) column was used with the mobile phase of 0.5 g/L sulfuric acid at 0.6 mL/min at 55℃. The yield (g/g) is defined as the ratio of accumulated succinate to consumed glycerol. Each value is an average of three parallel replicates and reported as mean ± standard deviation.

## Supplementary Information

**Additional file 1.** Effects of plasma treatment time on the lethal ratio of *A. succinogenes *NJ113.

## Data Availability

Not applicable.

## Abbreviations

MFC: Microbial fuel cells
ARTP: Atmospheric and room temperature plasma
NR: Neutral red
DMSO: Dimethyl sulfoxide
PEM: Proton exchange membrane
DCW: Dry cell weight
