# Supplementary material for: Microbial fuel cell-assisted utilization of glycerol for succinate production by mutant of Actinobacillus succinogenes
Source: Biotechnol Biofuels. 2021 Jan 15;14:23. doi: 10.1186/s13068-021-01882-5 (PMC7811241; doi:10.1186/s13068-021-01882-5)
Supplement: Supplementary file 1 — Additional file 1. Effects of plasma treatment time on the lethal ratio of A. succinogenes NJ113. [file 13068_2021_1882_MOESM1_ESM.docx]

**Microbial fuel cell assisted utilization of glycerol for succinate production by mutant of *Actinobacillus succinogenes***

Tianwen Zheng^1^, Bin Xu^1^, Yaliang Ji^1^, Wenming Zhang^1 2^, Fengxue Xin^1^ ^2^, Weiliang Dong^1^ ^2^, Ping Wei^1 2^, Jiangfeng Ma^1 2 *^, Min jiang^1 2^

^1^State Key Laboratory of Materials-Oriented Chemical Engineering, College of Biotechnology and Pharmaceutical Engineering, Nanjing Tech University, Nanjing, 211800, P. R. China

^2^Jiangsu National Synergetic Innovation Center for Advanced Materials (SICAM), Nanjing Tech University, Nanjing, 211800, P. R. China

*Corresponding author: Jiangfeng Ma. E-mail: [majiangfeng@njtech.edu.cn](mailto:majiangfeng@njtech.edu.cn)

Mailing address: State Key Laboratory of Materials-Oriented Chemical Engineering, College of Biotechnology and Pharmaceutical Engineering, Nanjing Tech University, Puzhu South Road 30#, Nanjing 211800, P. R. China.

Tel/Fax: 0086-25-58139927

**Table 1S** Carbon balance of conducted fermentation in MFC

| Strain | Initial glycerol  (mmol/L) | Glycerol consumption (mmol/L) | DCW (mmol/L) | Succinate (mmol/L) | Acetate (mmol/L) | Formate (mmol/L) | Carbon recovery ratio | Productivity (g/L/h) |
| --- | --- | --- | --- | --- | --- | --- | --- | --- |
| NJ113 | 107.61±3.80 | 53.64±3.47 | 8.87±0.40 | 26.93±1.95 | 19.32±2.16 | 23.03±2.82 | 0.94±0.05 | 0.04±0.01 |
|  | 117.71±3.88 ^a^ | 94.61±2.33 | 19.66±0.32 | 27.18±1.94 | 36.00±2.16 | 102.11±2.68 | 0.97±0.03 | 0.04±0.01 |
| JF1315 | 72.97±3.91 ^b^ | 57.88±1.52 | 22.98±0.81 | 37.18±0.93 | 20.48±1.00 | 13.03±0.22 | 1.00±0.08 | 0.06±0.01 |
|  | 89.26±3.91 | 89.26±3.91 | 34.68±3.23 | 44.12±0.93 | 38.47±1.33 | 0.00±0.00 | 0.91±0.01 | 0.11±0.01 |
|  | 296.34±1.41 | 296.34±1.41 | 52.42±3.23 | 202.56±0.68 | 19.48±12.99 | 57.14±2.39 | 0.85±0.05 | 0.39±0.01 |
|  | 644.37±2.39 | 409.60±0.76 | 10.08±0.81 | 149.04±7.45 | 75.27±2.33 | 322.62±16.95 | 0.76±0.07 | 0.23±0.01 |

Each value is an average of three parallel replicates and reported as mean ± standard deviation;

^a^: 10 g/L initial glycerol with 0.2 V potential applied to anode electrode.

^b^: 10 g/L initial glycerol with open circuit MFC without external resistance introduced.

**Figure 1S** Effects of plasma treatment time on the lethal ratio of *A. succinogenes* NJ113.
